# Intraocular Pressure and Retinal Nerve Fibre Layer Thickness Changes After Carotid Artery Stenting

**DOI:** 10.4274/tjo.67809

**Published:** 2017-08-15

**Authors:** Esra Biberoğlu, Muhsin Eraslan, Feyyaz Baltacıoğlu, İpek Midi

**Affiliations:** 1 Marmara University Faculty of Medicine, Department of Ophthalmology, İstanbul, Turkey; 2 Marmara University Faculty of Medicine, Department of Diagnostic and Interventional Radiology, İstanbul, Turkey; 3 Marmara University Faculty of Medicine, Department of Neurology, İstanbul, Turkey

**Keywords:** Carotid artery stenosis, stenting, color Doppler ultrasound, intraocular pressure, retinal nerve fiber layer thickness

## Abstract

**Objectives:**

The aim of this study was to evaluate intraocular pressure (IOP) and retinal nerve fiber layer (RNFL) changes in patients with carotid artery stenosis (CAS) after carotid artery stenting.

**Materials and Methods:**

This study was conducted as a cross-sectional, non-randomised clinical case series. Fifteen male patients (mean age: 63.6±9.1) with CAS and more than 70% carotid artery narrowing were included. All of the patients were followed in the department of neurology and were operated in the interventional radiology division. Eighteen healthy male subjects (mean age: 63.7±5.3) were included in the control group. All of the healthy subjects had a detailed ophthalmological examination and subjects with any chronic eye disease were excluded from the study. All of the participants had a detailed ophthalmological examination including tonometry using Goldmann applanation tonometry and RNFL analysis using optical coherence tomography (RTVue-100 5.1).

**Results:**

There were no ocular ischemic symptoms in any of the participants. The mean IOP value was 15.1±2.1 mmHg in the control group and 16.6±2.4 mmHg before stent implantation, 16.4±2.2 mmHg at 1 week after implantation, 16.6±2.5 mmHg at 1 month after implantation, and 16.7±2.9 mmHg at 3 months after implantation in the CAS group. Mean RNFL thickness was 105±6 µm in the control group; in the CAS group, mean RNFL thickness values were 98±27 µm before stent implantation and 103±11 µm, 101±10 µm, and 101±11 µm at 1 week, 1 month, and 3 months after stenting. There were no significant differences between the CAS group and control group regarding IOP and RNFL thickness values (p>0.05). IOP and RNFL thickness also did not show any statistically significant changes from preoperative measurements in 3 months postoperative follow-up in the CAS group (p>0.05).

**Conclusion:**

IOP and RNFL thickness remained unchanged after carotid stent implantation in carotid artery stenosis patients with no signs of ocular ischemic syndrome.

## INTRODUCTION

Carotid artery stenosis (CAS) is an important obstructive artery disease that can cause cranial ischemic infarction and stroke, and is the leading cause of ischemic stroke.^1^ The main goal in the treatment of CAS is to eliminate internal carotid artery (ICA) stenosis and the risk of embolism after carotid endarterectomy and carotid artery stenting (CS), as well as to increase retinal circulation.^2^ If ophthalmologic symptoms are assessed at an early stage, they can be managed prophylactically before reaching an irreversible stage and can be prevented at the onset, before the development of permanent blindness. At the same time, ophthalmologic findings can sometimes lead us to suspect CAS and facilitate the early diagnosis of stenosis before the patient develops symptoms like stroke.^3^ Patients who develop ocular ischemia may exhibit an increase in intraocular pressure (IOP) due to neovascularization; however, aqueous humor production decreases in some patients due to ciliary body ischemia, resulting in no IOP increase.^4^ Based on these data, in this study we aimed to compare changes in IOP and retinal nerve fiber layer (RNFL) thickness in patients who underwent CS.

## MATERIALS AND METHODS

The study included 15 male patients (mean age, 63.6±9.1 years) who were examined in the neurology department and diagnosed with CAS based on a finding of >70% narrowing of the carotid artery on color Doppler ultrasound (CDUS) and who underwent CS in the interventional radiology unit. Eighteen male participants (mean age, 63.7±5.3 years) were included as the control group. The study was conducted in accordance with the principles of the Declaration of Helsinki, informed consent forms were obtained from all participants, and approval was granted by the Ethics Committee for local clinical trials (protocol number: 09.2015.090 70737436-050.06.04).

**Figure 1:**
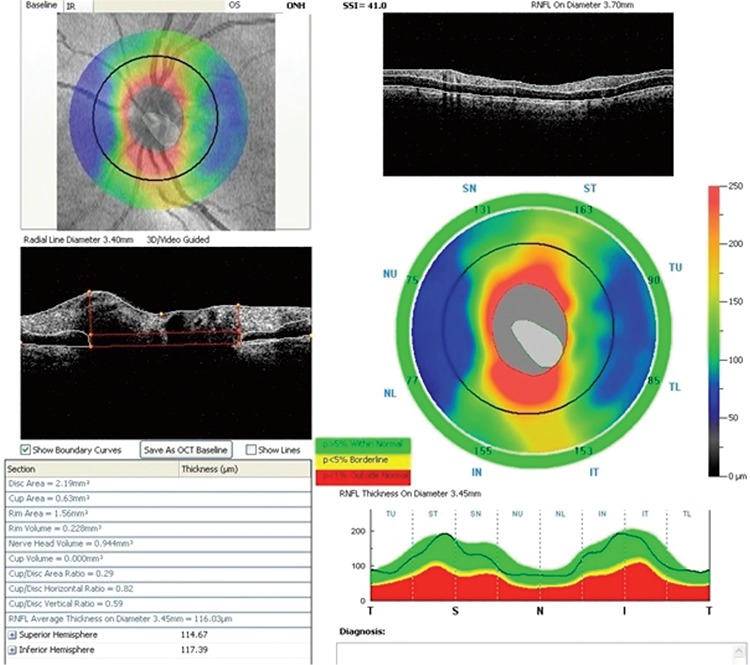
Optical coherence tomography output evaluating the average and superior and inferior hemisphere retinal nerve fiber layer thicknesses

The CS patients were asked specifically about hypertension, diabetes, and their alcohol and smoking history. Patients were ophthalmologically evaluated preoperatively and at postoperative 1 week, 1 month, and 3 months. All participants underwent a detailed eye examination. Best visual acuity was assessed using the Snellen chart. Snellen visual acuity values were converted to LogMAR values for statistical comparison. The anterior segment was assessed by slit-lamp examination. For IOP measurement, a mixture of 0.5% proparacaine and fluorescein was instilled into the eye and IOP was measured by Goldmann applanation tonometry. The average of three measurements was recorded. Optic nerve head (ONH) imaging was performed on all participants with the RTVue RT-100 spectral domain optical coherence tomography (Optovue Inc., Fremont, CA, USA) device in the ONH and 3D modes for glaucoma screening. The ONH program and three-dimensional disc program of the RT-100 were used for RNFL analysis. The ONH scanning protocol consisted of 12 radial images with a length of 3.7 mm, each making 455 scans transecting the center of the optic disc, and 13 concentric rings, each making between 425-965 scans and with diameters ranging from 1.3 to 4.9 mm. An RNFL thickness map was created from the RNFL thicknesses measured from the area within a 3.45 mm diameter of the disc center. The average and superior and inferior hemisphere RNFL thicknesses of the patients were evaluated (Figure 1).

The central corneal thickness (CCT) and axial lengths (AL) of all patients were measured by the same ophthalmologist using Haag-Streit International/LS 900 Lenstar. The lenses were evaluated for cataract after pupil dilation with tropicamide and phenylephrine eye drops. A detailed fundus examination including the entire retinal periphery was then conducted using a Volk SuperField NC lens. The eyes were evaluated for the presence of venous stasis retinopathy, iris neovascularization, glaucoma, optic nerve injury, vascular embolism, occlusion, and ocular ischemic syndrome (OIS).

Patients with visual acuity less than 6/10, spheric refraction exceeding -4 or +3 diopters (D), cylindrical refraction ≥±3 D, uveitis, glaucoma and retinal disease, optic disc damage, corneal and vitreal opacities, pupillary anomalies, history of ocular surgery other than phacoemulsification, cataract with NC<4, C<5, p<3 according to the LOCS II classification, systemic disease that may affect the measurements, or current drug treatment were not included in the study.

### Carotid Stenting Procedure

Before the procedure, patients were given detailed information about the treatment process and possible complications, and written consent forms were obtained. Patients whose procedures were planned were started on double antiaggregant therapy (75 mg clopidogrel + 100 mg acetylsalicylic acid twice daily) 3 days before the procedure. Patients who did not receive double antiaggregant therapy in advance and those undergoing emergency intervention were taken into surgery after being administered a loading dose of 450 mg clopidogrel. Hemogram, creatinine, and coagulation tests were performed as part of routine preparation. For these preoperative preparations, the patients were hospitalized the day before the procedure and monitored.

All procedures were conducted in an angiography unit equipped with a Siemens Artis Zee Bi-plain Angiography device, and all patients were monitored during CS. Electrocardiogram, oxygen saturation, and non-invasive arterial blood pressure were monitored during the procedure. In all patients, the right femoral artery was preferred as the entry site, and local anesthesia was applied to the area. At the beginning of the procedure, all patients were administered 5000 U of heparin intravenously.

After entering the femoral artery using the Seldinger technique, in 13 patients a 7F vascular sheath was inserted into the femoral artery and a 7F guiding catheter was advanced and positioned in the main carotid artery to be treated, while in 2 patients a 6F long vascular sheath was positioned directly in the main carotid artery. The guiding catheter and vascular sheath system was washed with pressurized isotonic serum throughout the procedure. Firstly, imaging of the neck and intracranial segments and intracranial branches of the carotid artery was performed to evaluate the hemodynamic changes that should occur in the intracranial vascular tree before and after the procedure and the possible presence of intracranial stenoses. Carotid angiography was then performed by administering contrast material via the catheter or vascular sheath placed in the main carotid artery to be stented, and a road map was obtained. Prior to the CS procedure, the guide wire of the protection filter was passed through the targeted stenosis and the filter was opened in a straight segment. Protection filters were routinely used in all patients; the Boston Scientific Filter Wire EZ Embolic Protection System was used in 13 patients and the Spider FX™ Embolic Protection Device was used in 2 patients. The monorail stent system was expanded above the guide wire carrying the filter, at the level of the lesion so as to encompass the area of stenosis determined using the road map. The Cristallo Ideale™ Carotid Stent System Self-Expanding stent was used in 11 patients and the Protege® RX Carotid Stent System Self-Expanding Nitinol stent was used in 4 patients. The stenosis was positioned high in 4 patients, so predilatation was performed with a 3x20 mm balloon prior to stenting in order to allow the stent to safely pass through at the level of the lesion. Postdilatation was performed on all patients after expanding the stent to allow it to reach its optimal span. Postdilatation was performed using a 6x20 mm balloon in 6 patients and a 5x20 mm balloon in 9 patients. After the procedure was completed, contrast material was administered to reevaluate the neck and intracranial arteries.

In the postoperative period, double antiaggregant therapy (75 mg clopidogrel + 100 mg acetylsalicylic acid daily) was administered to the patients for 3 months, to be followed by lifelong 100 mg acetylsalicylic acid prophylaxis.

### Statistical Analysis

SPSS (Statistical Package for Social Sciences) for Windows version 21.0 was used in statistical analysis of the study data. Mean ± standard deviation and percentage values were used for the descriptive statistics. Conformity of the data to normal distribution was assessed with the Kolmogorov-Smirnov test. Parametric tests were used to analyze numerical data with regular distribution, while nonparametric tests were used to analyze numerical data with irregular distribution.

Student’s t-test and the Mann-Whitney U test were used for intergroup comparisons and the paired intragroup comparisons of means. The chi-square test was used in the analysis of proportional data. Correlation analyses were done with the Pearson test. The statistical significance level was accepted as p<0.05 in analyses in which there were no variables from similar categories. When variables from the similar category were analyzed together, the significance level was decided according to the Bonferroni correction based on p<0.05.

## RESULTS

There were no significant differences between patients diagnosed with CAS and the control group in terms of mean age, spherical equivalents, visual acuity, CCT, or AL. A comparison of the patient and control groups’ general findings is provided in Table 1 and the other clinical features of the patients are summarized in Table 2.

None of the patients had ocular pain, retinal hemorrhage, or glaucoma. No complications were observed in any of the patients in follow-up examinations after stenting.

No statistically significant differences were observed when the preoperative, postoperative 1 week, postoperative 1 month, and postoperative 3 month IOP values of patients in the study group were compared with the control group (p<0.05) (Table 3).

There were also no statistically significant differences when the preoperative (n=15), postoperative 1 week (n=14), postoperative 1 month (n=8), and postoperative 3 month (n=10) IOPs of the patients were compared (p=0.963) (Table 4).

No statistically significant differences were observed when the preoperative, postoperative 1 week, postoperative 1 month, and postoperative 3 month RNFL values of patients diagnosed with CAS were compared with the control group (p<0.05) (Table 5).

## DISCUSSION

As the ophthalmic artery is a branch of the ICA, ocular involvement can occur in any case of ICA stenosis. Ocular involvement can range from transient unilateral acute blindness caused by emboli that break off from the atherosclerotic plaque in the stenosis, to chronic OIS due to persistent hypoperfusion or complete blindness due to occlusion of the central retinal artery or ophthalmic artery. OIS is characterized by ocular pain, decreased vision, patchy hemorrhages in the retina, and enlargement in the veins. Due to ocular hypoperfusion, any stenosis of the ICA can lead to ischemic retinopathy, neovascular glaucoma, ischemic optic neuropathy, retinal artery occlusions, cataract, and ocular hypotony. OIS and other findings also serve as indicators of cerebral ischemic disorders.

Microaneurysms, narrowing of the retinal arterioles, and venous dilation are observed on fundus examination due to decreased flow in the ophthalmic artery. When the ocular perfusion pressure lowers and approaches intraocular pressure, ischemia develops in both the posterior and anterior segments of the eye. Venous-stasis retinopathy limited to the posterior progresses to an OIS that also includes the anterior if the stenosis continues. Microproliferations that develop in the retinal vasculature and the iris form fibroadhesions. As a result, the anterior iridocorneal angle is occluded and intraocular pressure increases. If neovascularization is not treated, it can progress to neovascular glaucoma. After treating the carotid stenosis, these patients show improvements in their visual symptoms. In some patients, aqueous humor production decreases due to ciliary body ischemia, and there may be no increase in IOP.^4^ Rubeosis iridis is sometimes the only symptom associated with carotid stenosis.^5^ OIS is more common in carotid stenosis patients with weak collateral connections. The decrease in retrobulbar blood flow in patients with OIS can be demonstrated by CDUS. Retrograde flow, which is a predictor of high-grade carotid stenosis, can be seen in some patients. This retrograde flow leads to further exacerbation of ocular ischemia by further reducing retrobulbar blood flow due to the vascular steal phenomenon.^6,7,8^ Decreased vision and pain due to increased IOP may occur in ocular ischemia. In some cases, these symptoms may be the first clinical signs of CAS.

Hemispheric neurological symptoms, amaurosis fugax, and Hollenhorst plaques detected in ophthalmologic examination are findings that require imaging in the diagnosis of CAS. Retinal artery occlusion and ischemic optic neuropathy may also be related to carotid stenosis, as mentioned above. However, the predictive value of ocular findings in diagnosing stenosis is a subject of debate. Over 3 years, McCullough et al.^3^ evaluated 145 patients exhibiting these symptoms for carotid stenosis and found that amaurosis fugax had a 30% predictive value of clinical suspicion of carotid stenosis. Hollenhorst plaques were detected in 22 eyes, but only 4 of these patients had more than 60% stenosis in the carotid artery. In the same study, the authors reported that the presence of Hollenhorst plaques was found to be positively correlated with CAS at a rate of 18.2%, that the predictive values of other ocular symptoms such as ischemic optic neuropathy, retinal artery and vein occlusion, and optic atrophy were weak, and that stenosis was observed in one out of 5 patients who underwent CDUS after venous stasis retinopathy was detected, and its predictive value was 20%. Ultimately, they stated that Hollenhorst plaques and venous stasis retinopathy were of moderate value in the prediction of CAS.^3^ None of the patients in our study exhibited rubeosis iridis, neovascular glaucoma, or retinal artery or vein occlusion.

Studies show that the balance between ocular blood flow and IOP is important for ONH circulation. It has been demonstrated that as IOP increases, there is a decrease in the end diastolic flow rate and an increase in the resistance index of the arteries that supply the ocular structures, and end diastolic flow rate has been shown to negatively correlate with glaucoma progression.^9,10,11^ We also evaluated patients’ pre- and posttreatment IOP values in regards to possible CAS-related changes in ocular blood flow and the risk of glaucomatous optic neuropathy due to circulatory impairment, and observed no significant difference between the patient and control groups. In addition, no significant differences emerged when the pretreatment and posttreatment 1-week, 1-month, and 3-month IOP values of patients with CAS were compared with one another. This can be explained by the lack of patients with OIS in our patient group and the absence of ciliary body ischemia caused by stenosis, in which case there is no reduction in aqueous humor production.^12^

Sayin et al.^13^ compared the RNFL values of 25 patients diagnosed with CAS and 25 age-matched healthy control subjects and found no significant differences. In contrast, Pavan^14^ reported RNFL thinning in 8 CAS patients with over 70% stenosis. No difference was observed between our control group and patient group in terms of RNFL; however, there was also no change in the IOP and RNFL values of the patient group after stenting.

Ocular symptoms may be the first sign of serious carotid atherosclerotic disease. In this case, ophthalmologic examination is important for the prognosis of these patients. Patients at high risk for ischemic stroke can be referred for early intervention. In case of any retinal findings of OIS on ophthalmologic examination or a history of temporary monocular vision loss, the patient can be referred to the neurology clinic upon suspicion of CAS, thereby preventing ischemic neurological damage.

## CONCLUSION

In our study, there were no changes in baseline RNFL thickness and IOP values after stenting in patients with CAS who did not develop OIS. This finding must be supported by future studies including larger patient groups.

## Figures and Tables

**Table 1:**
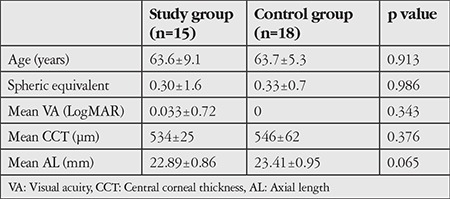
A comparison of the general findings of the patient and control groups

**Table 2:**
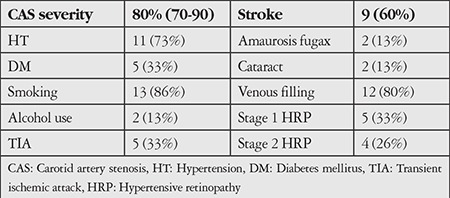
Clinical characteristics of the patient group

**Table 3:**
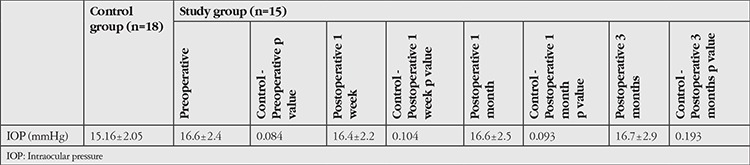
Comparison of intraocular pressure values between the control and study groups

**Table 4:**
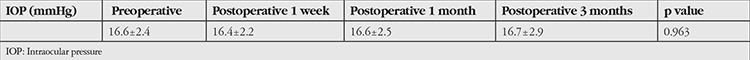
Comparison of pre- and postoperative intraocular pressures in the study group

**Table 5:**
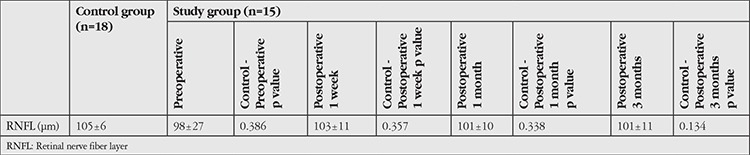
Comparison of optical coherence tomography parameters between the control and study groups
